# Is radiographic progression in modern rheumatoid arthritis trials still a robust outcome? Experience from tofacitinib clinical trials

**DOI:** 10.1186/s13075-016-1106-y

**Published:** 2016-09-23

**Authors:** Robert B. M. Landewé, Carol A. Connell, John D. Bradley, Bethanie Wilkinson, David Gruben, Sander Strengholt, Désirée van der Heijde

**Affiliations:** 1 Amsterdam Rheumatology Center, Amsterdam, The Netherlands; 2 Atrium Medical Center, Heerlen, The Netherlands; 3 Pfizer Inc, Groton, CT USA; 4 Pfizer Inc, Capelle aan den IJssel, The Netherlands; 5 Leiden University Medical Center, Leiden, The Netherlands

**Keywords:** Modified total Sharp score, Missing data, Outlier, Prognostic factors, Radiographic progression, Rheumatoid arthritis, Tofacitinib, Sensitivity analyses

## Abstract

**Background:**

The detection of statistically significant reductions in radiographic progression during clinical studies in patients with rheumatoid arthritis (RA) has become increasingly difficult over the past decade due to early-escape study designs and declining rates of progression in control-group patients. We investigated the impact of extremes of radiographic data (outliers) and baseline prognostic factors on detection of treatment effects, to provide guidance on future analysis of joint structural data in RA clinical trials.

**Methods:**

Data were from two, phase 3, randomized, double-blind, placebo-controlled trials of tofacitinib in adult patients with moderate to severe RA: ORAL Scan (NCT00847613) and ORAL Start (NCT01039688). These studies detected significant reductions in radiographic progression with tofacitinib 10 mg twice daily (BID) plus background methotrexate (ORAL Scan), and with tofacitinib 5 or 10 mg BID as monotherapy (ORAL Start). We evaluated mean changes from baseline in van der Heijde modified total Sharp score (mTSS) at month 6 and month 12, using analysis of covariance (ANCOVA). A trimmed analysis was used to deal with extremes of data. The impact of baseline prognostic factors on radiographic progression was evaluated using ANCOVA to analyze the mean change from baseline in mTSS for each factor in turn.

**Results:**

The analysis included data from 720 patients from ORAL Scan and 880 patients from ORAL Start. Trimmed analyses were unbiased for the true mean estimate and enabled us to remove the effect of influential extreme observations in the data set. Almost all patients had at least one poor prognostic factor at baseline (e.g., high level of disease activity, or positive for rheumatoid factor). The strongest predictor of treatment effect was the severity of radiographic damage at baseline.

**Conclusions:**

A trimmed analysis can establish whether any significant inhibition of structural damage is being driven by extremes of data, and should be one of the sensitivity analyses of choice for structural data in RA clinical trials. Furthermore, analysis of radiographic data based on baseline prognostic factors may reveal increased treatment effects. Application of these methods to analysis of radiographic data from clinical trials in patients with RA, allows a more complete interpretation of data.

**Trial registration:**

Clinicaltrials.gov NCT00847613 (registered 17 February 2009) and NCT01039688 (registered 23 December 2009)

**Electronic supplementary material:**

The online version of this article (doi:10.1186/s13075-016-1106-y) contains supplementary material, which is available to authorized users.

## Background

During the past decade, radiographic progression rates observed in rheumatoid arthritis (RA) clinical trials have gradually decreased [1–3]. The ethical necessity for the placebo treatment periods of RA trials to be of short duration (typically 12–16 weeks) [4, 5], has resulted in the use of early-escape trial designs to minimize exposure to placebo [5–8]. This presents methodological challenges to the demonstration of treatment effect and magnitude of effect, as low rates of radiographic progression in control groups may impact upon the statistical power of such trials to detect a true RA treatment effect [3]. Moreover, since there is also a requirement for trials of RA therapies to provide long-term efficacy data (≥1 year) [4], researchers may have no alternative but to extrapolate efficacy data, including structural efficacy. Such extrapolations tend to result in wider confidence intervals (CIs) [9] and increase the likelihood that plots of data over time for the reference and comparator arms will cross, thus making the detection of true treatment differences more difficult.

From a methodological perspective, increasing the number of patients and/or the inclusion of patients with RA who are at high risk for radiographic progression in clinical trials, may increase the power of a trial to detect true treatment effects. However, as diagnoses of RA may now be made early in the disease course, and as initial treatments become more aggressive, rapid development of erosions is less likely to be seen. Based on the above considerations, it is important to confirm that a treatment effect on radiographic progression in controlled clinical trials – such as between-group differences in the change from baseline in the van der Heijde modified total Sharp score (mTSS) [10] – represents a robust outcome. Sensitivity analyses can be used to confirm the credibility of clinical trial findings [11] and further explore results of marginal statistical significance, and trends that are not statistically significant.

In this article, we explore two distinct post hoc methodologies that may enhance the ability to demonstrate a true treatment effect on structural progression in RA clinical trials, including sensitivity to the effects of extremes of data (outliers) using a trimmed analysis approach, and the impact of prognostic factors on the ability to detect a treatment effect.

We have used two recent phase 3 randomized controlled trials (RCTs) of tofacitinib, an oral Janus kinase inhibitor for the treatment of RA, as examples for radiographic progression: ORAL Scan (NCT00847613) [12] and ORAL Start (NCT01039688) [13]. Published results of the ORAL Scan study (conducted in patients receiving background methotrexate [MTX]) showed that tofacitinib 10 mg twice daily (BID) was effective in reducing radiographic progression versus placebo at month 6 (*p* ≤ 0.05) in the primary analysis [12]. Tofacitinib 5 mg BID was associated with numerical improvements in mTSS, although statistical significance was not reached (*p* = 0.0792) [12], and results observed using rank analysis as a sensitivity measure [14] were inconsistent. In the ORAL Start study, in which tofacitinib was administered as monotherapy, both tofacitinib 5 and 10 mg BID were associated with statistically significant reductions in radiographic progression versus MTX at month 6 (*p* < 0.001 for both comparisons) in the primary analysis [13], which was confirmed by rank analysis. Analyses of the percentage of patients with radiographic progression have previously been published for both studies [12, 13]. Here we discuss methodologies applicable to analysis and interpretation of mean changes in mTSS.

## Methods

### Designs of the phase 3 ORAL Scan and ORAL Start clinical trials

Full details of ORAL Scan and ORAL Start, including patient populations, have been reported elsewhere [12, 13]. Both studies were double-blind, parallel-group trials of 24 months’ duration, and were designed to evaluate the efficacy and safety of tofacitinib in adult patients (aged ≥18 years) with active moderate to severe RA [12, 13]. Patients had either an inadequate response to MTX (ORAL Scan) [12] or were MTX-naïve (ORAL Start) [13].

In ORAL Scan, patients were randomized (4:4:1:1) to tofacitinib 5 mg BID, tofacitinib 10 mg BID, placebo advanced to tofacitinib 5 mg BID, and placebo advanced to tofacitinib 10 mg BID. All patients received stable background MTX. Patients randomized to placebo were advanced to tofacitinib 5 or 10 mg BID, according to the randomized treatment regimen, at either month 3 (non-responders; did not achieve ≥20 % improvement in swollen and tender joint counts) or month 6 (all other patients). Due to the early-rescue study design, there were no patients receiving placebo beyond month 6.

In ORAL Start, patients were randomized (2:2:1) to receive tofacitinib as monotherapy (5 or 10 mg BID), or MTX (10 mg/week, titrated up to 20 mg/week by week 8). Patients received their randomized treatment as per protocol (tofacitinib or MTX) throughout their participation in the 24-month study; there was no rescue of inadequate responders in this study.

### Radiographic scoring

Radiographs of both hands and feet were taken at baseline, then at month 3 in non-responders (ORAL Scan only) and at months 6, 12, and month 24 (or end of study). The van der Heijde mTSS was used to assess radiographic progression [10]. Radiographs were graded by two independent, blinded readers who viewed the entire set of radiographs for a patient in a single reading session (concealed time order). All error (e.g., measurement error) was divided randomly and symmetrically in both tails of the distribution so that the sum of all error could be expected to be zero. Adjudication was performed in the event of any large discrepancy in mTSS between the two independent readers.

### Statistical analysis of radiographic progression

The primary efficacy analyses of ORAL Scan and ORAL Start have been published previously [12, 13]. The primary efficacy analyses included progression in radiographic scores measured by mean change from baseline in mTSS at month 6, based on the month 12 interim analysis [12, 13]. The current analysis included radiographic progression data at month 6 and month 12 from the 12-month interim analysis (some values may differ from the final, locked study databases). Both the primary analyses and the current analysis included all randomized patients who received ≥1 dose of study medication and who had a baseline measurement and at least one subsequent measurement.

Radiographic progression, measured by mean change from baseline in mTSS at month 6 and month 12, was analyzed using analysis of covariance (ANCOVA), with a least squares (LS) approach to solve values. The ANCOVA model included treatment, geographic location and baseline mTSS value, and duration of RA (for ORAL Start), as covariates. Linear extrapolation was used to impute missing values. Where month 6 data were not available, month 3 data were extrapolated to month 6; month 3 data are not reported here.

After the publication of ORAL Start [13], one of its study sites (eight patients randomized) was found to be non-compliant with study procedures and those patients have been removed from the efficacy analyses presented here.

### Sensitivity analysis

To investigate the trimmed analysis approach to deal with extremes of mTSS values, we used data from the primary analyses of mean change from baseline in mTSS at months 6 and 12. Trimmed analysis involved assignment of a percentile rank to data for mean change from baseline in mTSS at months 6 and 12 for each treatment group. A fixed percentage of data points were then removed in equal amounts from the top and bottom ranks of each treatment group (‘trimming’), thus 1 % trimming resulted in deleted observations from <1st percentile and >99th percentile, 2 % of data being deleted in total. ANCOVA was applied to the trimmed data set, with the process subsequently repeated in increments of 1 % up to 10 %, and the analysis for each of the trimmed data sets was compared.

To investigate whether the presence at baseline of poor prognostic factors for radiographic progression is associated with a higher treatment effect, we performed a post hoc analysis of mTSS data from ORAL Scan and ORAL Start. We selected a number of prognostic factors that are known to predict radiographic progression in RA, including erythrocyte sedimentation rate (ESR), anti-cyclic citrullinated peptide positivity (CCP+), rheumatoid factor positivity (RF+), C-reactive protein (CRP) levels, erosion score, and mTSS score at baseline [15–20]. ANCOVA was used to analyze the mean change from baseline in mTSS at month 6 and month 12 for each of the subsets for every prognostic factor in turn. An additional analysis of baseline mTSS subsets by categorization according to tertiles (i.e., three subsets, with patients with the highest baseline mTSS values in the top third of the sample at greatest risk of progression) was performed to investigate any ‘dose effect’ of baseline structural damage on the observable treatment difference.

## Results

In total, 720 patients in ORAL Scan and 880 patients in ORAL Start had at least one post-baseline radiograph and were included in the analysis. In ORAL Scan, radiographs from 706 patients were available for analysis at month 6 (tofacitinib 5 mg BID, N = 277; tofacitinib 10 mg BID, N = 290; placebo, N = 139) and 720 at month 12 (tofacitinib 5 mg BID, N = 286; tofacitinib 10 mg BID, N = 295; placebo, N = 139). In ORAL Start, radiographs from 875 patients were available for analysis at month 6 (tofacitinib 5 mg BID, N = 344; tofacitinib 10 mg BID, N = 367; MTX, N = 164) and 879 at month 12 (tofacitinib 5 mg BID, N = 343; tofacitinib 10 mg BID, N = 368; MTX, N = 168).

## Impact of outliers on the ability to detect a treatment effect

**Fig. 1:**
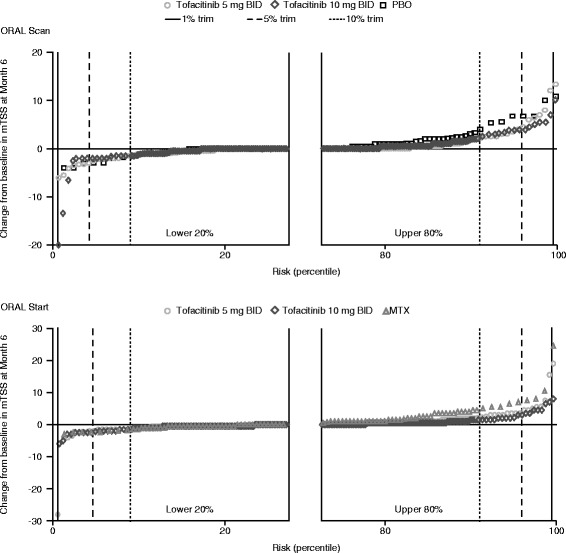
Cumulative probability plots showing individual patient changes from baseline in mTSS at month 6. *BID* twice daily, *mTSS* van der Heijde modified total Sharp score, *MTX* methotrexate, *PBO* placebo

Change from baseline in van der Heijde mTSS is a common measure of progression of joint destruction in patients with RA. Typically, as shown by cumulative probability plots for the distribution for changes from baseline in mTSS at month 6 (primary analysis) in ORAL Scan and ORAL Start, a large proportion of patients have little or no change in mTSS, with fewer patients having larger changes (Fig. 1). Where changes, either positive or negative, are extreme, this may result from a combination of true effect and measurement errors [3]. While such extreme data points have minimal influence on treatment effects, they do contribute to variability (higher standard deviations) and may, therefore, jeopardize statistical comparisons. Indeed, statistical analyses of the mean change from baseline in mTSS using ANCOVA may be influenced by extreme values. Although rank analysis is a commonly used approach to remove the influence of extreme values [21], it can reduce sensitivity for detecting differences in mTSS values, particularly when the mean rate of progression is low [14]. The effects of extreme values can be investigated by ‘trimming’ to systematically remove increasing proportions of extreme values from both ends of the mTSS distribution curve [14].

### Sensitivity analysis using a trimmed analysis approach

The cumulative probability plots shown in Fig. 1 demonstrate how 1 %, 5 %, and 10 % of trimming of data will lead to different distributions of change from baseline in mTSS.

**Fig. 2:**
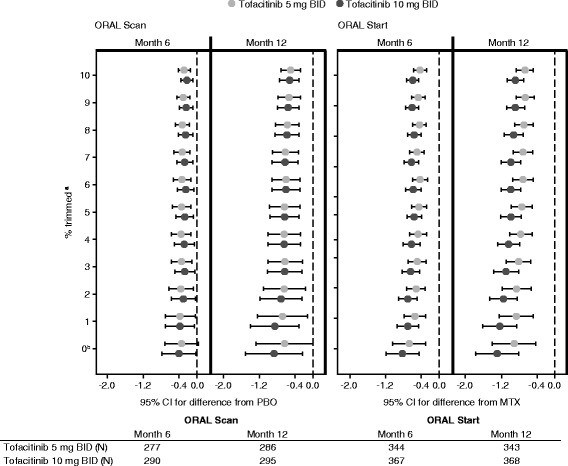
Trimmed analysis of differences from comparator in mTSS at month 6 and month 12 in ORAL Scan and ORAL Start. 0 % trimming represents the primary analysis. ^a^Percentage of data excluded; LS mean differences from PBO (ORAL Scan) or MTX (ORAL Start) with 95 % CIs of each tofacitinib group vs comparator are presented; a CI that does not contain 0 indicates that the difference is statistically significant (*p* < 0.05). *BID* twice daily, *CI* confidence interval, *LS* least squares, *mTSS* van der Heijde modified total Sharp score, *MTX* methotrexate, *N* number of patients eligible for analysis, *PBO* placebo

In ORAL Scan, untrimmed data at month 6 (equivalent to the primary analysis) and month 12 showed that patients treated with tofacitinib 10 mg BID, but not tofacitinib 5 mg BID, had significantly less radiographic progression from baseline versus placebo (Fig. 2; Table S1 in Additional file 1). For both tofacitinib 5 and 10 mg BID, statistical significance versus placebo (CI <0; *p* ≤ 0.05 [not corrected for multiple comparisons]) was achieved for both tofacitinib 5 and 10 mg BID at months 6 and 12 at 1 % trimming, and with further trimming, with mean values stable from ≥3 % trimming (Fig. 2; Table S1 in Additional file 1). Thus, consistency was observed between the primary analysis and trimmed data for the tofacitinib 10 mg BID dose in ORAL Scan (Fig. 2), indicating that the results were not dependent on extreme data. However, such consistency was not observed between the untrimmed and trimmed ORAL Scan data sets for tofacitinib 5 mg BID, suggesting that the primary analysis for tofacitinib 5 mg BID in this study was influenced by extreme values.

In ORAL Start, untrimmed data at month 6 (equivalent to the primary analysis) and month 12 demonstrated that both tofacitinib doses inhibited progression of structural damage compared with MTX (Fig. 2; Table S1 in Additional file 1). The trimmed analysis for ORAL Start showed that statistical significance (CI <0; *p* ≤ 0.05) was maintained for both doses of tofacitinib with ≥1 % data trimming and, especially for month 6, the upper limit of the CI (denoting a conservative estimate of the efficacy vs MTX) remained stable for tofacitinib 5 and 10 mg BID from ≥3 % trimming (Fig. 2; Table S1 in Additional file 1). Thus, trimming did not influence the statistical comparisons between tofacitinib 5 or 10 mg BID and MTX, confirming the stability of the primary analysis.

To the best of our knowledge, the use of a trimmed analysis approach to correct for extremes of joint structural data is unique in the RA setting. However, trimming has been applied in modelling the length of pediatric hospital stay, where explicit values were trimmed, rather that the approach taken here to trim specific percentages [22]. Our findings show that trimmed analysis represents a useful means of checking the contribution of extremes of structural data in patients with RA. This approach represents a conceptual bridge between a rank analysis and ANCOVA, and is unbiased for the true mean estimate, while removing the effect of influential observations in any one data set. Indeed, trimmed analysis gives improved insight into the influence of extreme values and should be considered as one of the sensitivity analyses of choice for structural data.

## Impact of prognostic factors on the ability to detect a treatment effect

In the absence of radiographic progression in the control group, it is not possible to demonstrate that an effective drug delays structural joint damage, and in such situations, a clinical trial with mean change from baseline in mTSS as the primary outcome will fail. Prognostic factors such as ESR, CCP seropositivity, RF seropositivity, C-reactive protein levels, and early evidence of erosions are known to be independently predictive of poor outcomes in patients with RA [23]. In addition, baseline mTSS score is predictive of joint damage progression [24] and could be used to identify patients who are more likely to experience progression, including rapid progression, which may allow a treatment effect to be more readily discerned.

### Analyses of mTSS data in high-risk subgroups

The proportion of patients in the ORAL Scan and ORAL Start studies who had poor prognostic factors at baseline is shown in Table 1.

**Table 1:** Prevalence of poor prognostic factors at baseline in ORAL Scan and ORAL Start

	ORAL Scan	ORAL Start
	(N = 720)	(N = 880)
Patients with poor prognostic factor, n/N (%)		
CCP+	600/718 (83.6)	737/880 (83.8)
DAS28-4(ESR) >5.1	635/715 (88.8)	822/879 (93.5)
RF+	546/718 (76.0)	726/880 (82.5)
CRP+ (>7 mg/L)	416/720 (57.8)	592/880 (67.3)
Erosion score ≥3	460/720 (63.9)	403/880 (45.8)
Baseline mTSS > median^a^	360/720 (50.0)	433/880 (49.2)
Number of poor prognostic factors present at baseline, n/N (%)		
≥ 1	719/720 (99.9)	878/880 (99.8)
≥ 2	689/720 (95.7)	847/880 (96.3)
≥ 3	627/720 (87.1)	780/880 (88.6)
≥ 4	504/720 (70.0)	625/880 (71.0)
≥ 5	315/720 (43.8)	368/880 (41.8)
6	163/720 (22.6)	215/880 (24.4)

There were two patients in the ORAL Scan study and two patients in the ORAL Start study who had no poor prognostic factors at baseline

*CCP* cyclic citrullinated peptide, *CRP* C-reactive protein, *DAS28-4*(*ESR*) Disease Activity Score in 28 joints (erythrocyte sedimentation rate), *mTSS* van der Heijde modified total Sharp score, *RF* rheumatoid factor

^a^Median baseline mTSS value was 13.1 for ORAL Scan and 4.0 for ORAL Start

**Fig. 3:**
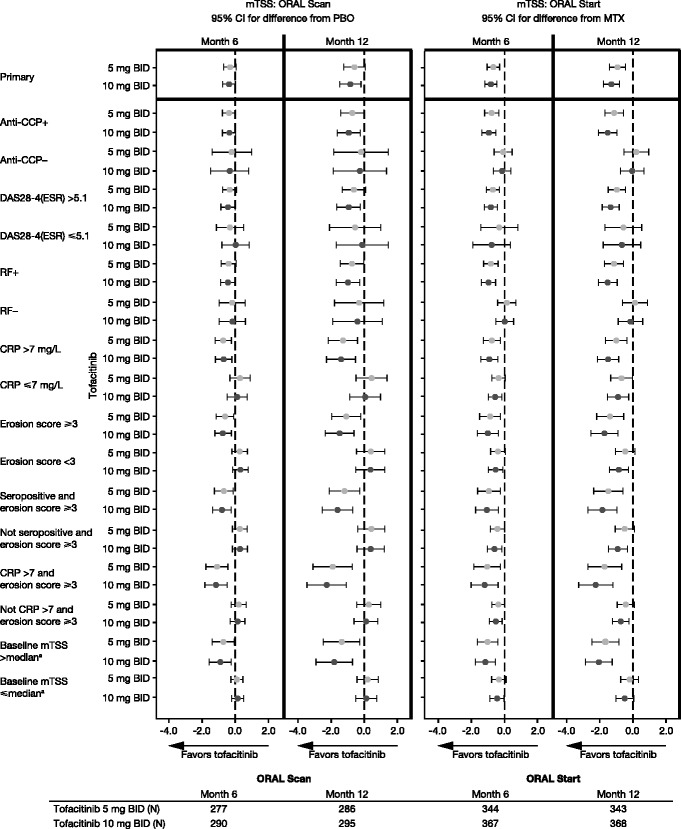
Differences from comparator in mTSS (month 6 and month 12) according to baseline prognostic factors. ^a^Median baseline mTSS value was 13.1 for ORAL Scan and 4.0 for ORAL Start. LS mean differences from placebo (ORAL Scan) or MTX (ORAL Start) with 95 % CIs of each tofacitinib group vs comparator are presented; a CI that does not contain 0 indicates that the difference is statistically significant (*p* < 0.05). The ANCOVA model used was the same for each subgroup and included effects for treatment, geographic location, and baseline value of mTSS. The ANCOVA model for the ORAL Start study initially included a categorical variable for duration of RA at baseline. Missing values were imputed by linear extrapolation. Across both studies and tofacitinib doses the number of patients in each subgroup ranged from: 234–298 for anti-CPP+; 42–70 for anti-CCP-; 250–346 for DAS28-4(ESR) >5.1; 22–34 for DAS28-4(ESR) ≤5.1; 206–301 for RF+; 60–72 for RF-; 160–237 for CRP >7 mg/L; 109–131 for CRP ≤7 mg/L; 101–205 for erosion score <3; 162–193 for erosion score ≥3;140–177 for seropositive and erosion score ≥3; 116–164 for not seropositive and erosion score ≥3; 108–122 for CRP >7 and erosion score ≥3; 169–258 for not CRP >7 and erosion score ≥3; 137–181 for baseline mTSS > median; and 140–187 for baseline mTSS ≤ median. *ANCOVA* analysis of covariance, *BID* twice daily, *CCP* cyclic citrullinated peptide, *CI* confidence interval, *CRP* C-reactive protein, *DAS28-4(ESR)* Disease Activity Score in 28 joints (erythrocyte sedimentation rate), *LS* least squares, *mTSS* van der Heijde modified total Sharp score, *MTX* methotrexate, *PBO* placebo, *RF* rheumatoid factor

Almost all patients in both trials had at least one poor prognostic factor at baseline (Table 1). This finding was as expected, as the majority of patients included in phase 3 RA clinical trials were RF+ and/or CPP+, and had a high level of disease activity. Moreover, a high percentage of patients had two or more poor prognostic factors (Table 1). Although we considered the possibility that such patients may be less responsive to treatment (i.e., that rapid progression would be a negative predictor of response) in general, the subsets of patients with poor prognostic factors showed more pronounced treatment effects, in terms of change from baseline in mTSS, with tofacitinib 5 and 10 mg BID (Fig. 3). However, not all prognostic factors had equal impact. Although RF and CCP seropositivity and CRP level were significant predictors of efficacy and treatment effect, the strongest individual predictors of a treatment effect were baseline mTSS or baseline erosion score. Combining erosion score with CCP and/or RF seropositivity did not attenuate this effect, however combining erosion score with CRP level did appear to increase the predictive effect (Fig. 3).

**Fig. 4:**
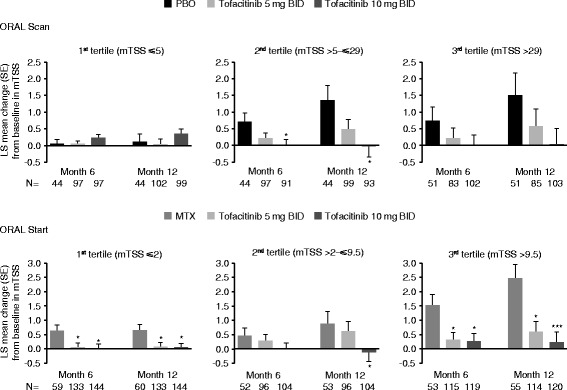
Change from baseline in mTSS according to baseline mTSS-defined tertiles for ORAL Scan and ORAL Start. ^*^ *p* ≤ 0.05; ^**^ *p* ≤ 0.001; and ^***^ *p* < 0.0001 vs PBO or MTX. In post hoc analyses, presented values are descriptive. Data presented in Fig. 3 as a Forest plot are presented here as bar graphs, showing that moving from a lower to a higher tertile category indicates an increase in treatment effect. *BID* twice daily, *LS* least squares, *mTSS* van der Heijde modified total Sharp score, *MTX* methotrexate, *PBO* placebo, *SE* standard error

Analysis of baseline mTSS according to tertiles showed that, in both ORAL Scan and ORAL Start, a larger treatment effect was observed with increased baseline structural damage (baseline mTSS third tertile vs first tertile). Mean increases exceeding 0.5 in placebo and MTX groups were matched with much less pronounced progression and even zero and negative progression, in the tofacitinib groups (Fig. 4). However, the tertile analysis did not provide consistent statistically significant differences between tofacitinib and comparator, probably as a result of the small patient numbers in each group.

This approach demonstrates how an indirect enrichment of the data through post hoc analyses might help to differentiate responders from non-responders, while allowing a realistic enrollment of patients in a timely manner.

Various published studies in RA have investigated the effects of poor prognostic factors on clinical efficacy. However, only a few analyses specifically address the effects of baseline prognostic markers on radiographic outcomes [19, 20, 25, 26]. We identified several recent studies that investigated the effect of biologic disease-modifying antirheumatic drugs on joint structural preservation in patients with RA, although few used baseline radiographic data as a prognostic marker. One such study was C-OPERA, which evaluated radiographic progression in MTX-naïve patients with early RA who received certolizumab pegol with MTX [27]. Treatment effect was analyzed according to CCP seropositivity (an inclusion criterion for the study), RF seropositivity, and presence of bone erosions at baseline [27]. Inhibition of radiographic progression was assessed at weeks 24 and 52 using the van der Heijde mTSS. In agreement with the findings of the present analysis, the authors concluded that treatment with certolizumab was more likely to prevent joint damage in patients with higher disease activity at baseline or with early evidence of bone erosions [27].

The C-OPERA study described above [27] is an example of study population enrichment for individuals at high risk of radiographic progression. While it follows that it may be desirable to enrich study populations for other poor prognostic factors, as defined here and in European League Against Rheumatism (EULAR) recommendations [23], the optimum number of baseline risk factors is open to debate.

## Conclusions

Demonstration of a reduction in joint structural damage via measurement of radiographic progression in RA is challenging due to the limited duration of placebo control, and a low level of progression observed in the placebo group – which patients receive in addition to background therapy – and limitations of current analytical methods [28]. In the present analysis, we explored several different methodologies to correct for these effects, taking into consideration the effects of extremes of data, and baseline prognostic factors for radiographic progression. These methodologies are well described in published literature, although infrequently used in previous evaluations of radiographic progression in RA.

The trimmed analysis approach described here allowed us to visualize the effect of potential outliers, with stable mean values providing assurance of a real treatment effect versus comparators. Furthermore, analysis of high-risk subsets of patients based on known prognostic factors increased the observable treatment difference. If we wish to detect and demonstrate true treatment differences in trials that have a focus on structural preservation in patients with RA, then these factors should be taken into account at the trial design stage.

In conclusion, using a trimmed analysis approach can establish whether or not significant inhibition of structural damage is driven by extremes of data (outliers), and that analysis of radiographic data based on prognostic factors at baseline may reveal increased treatment effects. Applying these analytic methodologies to the assessment of radiographic progression allows a more complete interpretation of data and verification of radiographic results reported in RA RCTs, which can be difficult to evaluate accurately in current clinical trials.

## Additional file

Additional file 1: Table S1. Trimmed analysis of data for mTSS change from baseline at month 6 and month 12 in ORAL Scan and ORAL Start. The table provides least squares mean changes from baseline at month 6 and month 12, and treatment differences versus comparator with 95 % CIs for both the ORAL Scan and ORAL Start studies. (DOCX 15 kb)

## Abbreviations

ANCOVA: analysis of covariance
BID: twice daily
CCP: cyclic citrullinated peptide
CI: confidence interval
CRP: C-reactive protein
DAS28-4(ESR): Disease Activity Score in 28 joints (erythrocyte sedimentation rate)
ESR: erythrocyte sedimentation rate
LS: least squares
mTSS: van der Heijde modified total Sharp score
MTX: methotrexate
N: number of patients eligible for analysis
PBO: placebo
RA: rheumatoid arthritis
RCT: randomized controlled trial
RF: rheumatoid factor
SE: standard error
