# Supplementary material for: Is radiographic progression in modern rheumatoid arthritis trials still a robust outcome? Experience from tofacitinib clinical trials
Source: Arthritis Res Ther. 2016 Sep 23;18:212. doi: 10.1186/s13075-016-1106-y (PMC5034418; doi:10.1186/s13075-016-1106-y)
Supplement: Additional file 1: Table S1. — Trimmed analysis of data for mTSS change from baseline at month 6 and month 12 in ORAL Scan and ORAL Start. The table provides least squares mean changes from baseline at month 6 and month 12, and treatment differences versus comparator with 95 % CIs for both the ORAL Scan and ORAL Start studies. (DOCX 15 kb) [file 13075_2016_1106_MOESM1_ESM.docx]

**ADDITIONAL FILE 1**

**Supplementary Table 1.** Trimmed analysis of data for mTSS change from baseline at Month 6 and Month 12 in ORAL Scan and ORAL Start

| **Trimming,%** | **Treatment** | **mTSS change from baseline at 6 months in ORAL Scan** | | **mTSS change from baseline at  12 months in ORAL Scan** | | **mTSS change from baseline at 6 months in ORAL Start** | | **mTSS change from baseline at  12 months in ORAL Start** | |
| --- | --- | --- | --- | --- | --- | --- | --- | --- | --- |
|  |  | **LS mean (±SE)** | **Difference from PBO (95% CI)** | **LS mean (±SE)** | **Difference from PBO (95% CI)** | **LS mean (±SE)** | **Difference from MTX (95% CI)** | **LS mean (±SE)** | **Difference from MTX (95% CI)** |
| 0 | Tofacitinib 5 mg BID | 0.12 (0.12) | -0.34 (-0.73, 0.04) | 0.29 (0.19) | -0.63 (-1.27, 0.02) | 0.19 (0.12) | -0.66 (-1.04, -0.28)*** | 0.35 (0.15) | -0.91 (-1.40, -0.42)*** |
|  | Tofacitinib 10 mg BID | 0.06 (0.11) | -0.40 (-0.79, -0.02)* | 0.05 (0.19) | -0.87 (-1.51, -0.23)** | 0.04 (0.11) | -0.81 (-1.18, -0.43)*** | -0.01 (0.15) | -1.28 (-1.76, -0.79)*** |
|  | PBO^a^ or MTX^b^ | 0.47 (0.16) | - | 0.92 (0.28) | - | 0.85 (0.16) | - | 1.27 (0.21) | - |
| 1 | Tofacitinib 5 mg BID | 0.08 (0.10) | -0.37 (-0.69, -0.04)* | 0.23 (0.17) | -0.68 (-1.23, -0.12)* | 0.16 (0.08) | -0.53 (-0.78, -0.29)*** | 0.27 (0.12) | 0.86 (-1.24, -0.48)*** |
|  | Tofacitinib 10 mg BID | 0.08 (0.10) | -0.37 (-0.69, -0.05)* | 0.06 (0.17) | -0.85 (-1.40, -0.30)** | 0.01 (0.07) | -0.69 (-0.93, -0.45)*** | -0.09 (0.11) | -1.22 (-1.60, -0.84)*** |
|  | PBO^a^ or MTX^b^ | 0.45 (0.14) | - | 0.91 (0.24) | - | 0.70 (0.10) | - | 1.13 (0.17) | - |
| 2 | Tofacitinib 5 mg BID | 0.05 (0.08) | -0.35 (-0.62, -0.07)* | 0.15 (0.14) | -0.64 (-1.11, -0.18)** | 0.16 (0.07) | -0.51 (-0.72, -0.30)*** | 0.21 (0.10) | -0.84 (-1.16, -0.52)*** |
|  | Tofacitinib 10 mg BID | 0.10 (0.08) | -0.30 (-0.57, -0.03)* | 0.09 (0.14) | -0.71 (-1.17, -0.24)** | -0.02 (0.06) | -0.69 (-0.90, -0.48)*** | -0.09 (0.10) | -1.14 (-1.45, -0.82)*** |
|  | PBO^a^ or MTX^b^ | 0.40 (0.12) | - | 0.80 (0.20) | - | 0.67 (0.09) | - | 1.05 (0.14) | - |
| 3 | Tofacitinib 5 mg BID | 0.02 (0.07) | -0.34 (-0.57, -0.10)** | 0.13 (0.12) | -0.62 (-1.01, -0.23)** | 0.14 (0.06) | -0.48 (-0.68, -0.28)*** | 0.20 (0.09) | -0.80 (-1.09, -0.52)*** |
|  | Tofacitinib 10 mg BID | 0.09 (0.07) | -0.27 (-0.50, -0.03)* | 0.12 (0.11) | -0.63 (-1.02, -0.25)** | -0.01 (0.06) | -0.63 (-0.83, -0.43)*** | -0.08 (0.08) | -1.08 (-1.36, -0.81)*** |
|  | PBO^a^ or MTX^b^ | 0.36 (0.10) | - | 0.75 (0.17) | - | 0.62 (0.09) | - | 1.00 (0.12) | - |
| 4 | Tofacitinib 5 mg BID | 0.02 (0.07) | -0.35 (-0.57, -0.12)** | 0.09 (0.11) | -0.64 (-1.01, -0.27)*** | 0.11 (0.06) | -0.46 (-0.65, -0.27)*** | 0.19 (0.08) | -0.75 (-1.01, -0.50)*** |
|  | Tofacitinib 10 mg BID | 0.09 (0.07) | -0.28 (-0.50, -0.06)* | 0.09 (0.11) | -0.64 (-1.01, -0.27)*** | -0.03 (0.06) | -0.60 (-0.79, -0.42)*** | -0.08 (0.08) | -1.03 (-1.28, -0.77)*** |
|  | PBO^a^ or MTX^b^ | 0.37 (0.09) | - | 0.73 (0.16) | - | 0.57 (0.08) | - | 0.95 (0.11) | - |
| 5 | Tofacitinib 5 mg BID | 0.01 (0.06) | -0.34 (-0.54, -0.13)** | 0.09 (0.10) | -0.63 (-0.97, -0.30)*** | 0.10 (0.06) | -0.44 (-0.61, -0.26)*** | 0.17 (0.07) | -0.74 (-0.97, -0.51)*** |
|  | Tofacitinib 10 mg BID | 0.08 (0.06) | -0.27 (-0.47, -0.07)** | 0.09 (0.10) | -0.63 (-0.96, -0.29)*** | -0.01 (0.05) | -0.55 (-0.72, -0.37)*** | -0.06 (0.07) | -0.97 (-1.20, -0.74)*** |
|  | PBO^a^ or MTX^b^ | 0.35 (0.09) | - | 0.72 (0.14) | - | 0.53 (0.08) | - | 0.91 (0.10) | - |
| 6 | Tofacitinib 5 mg BID | 0.01 (0.06) | -0.33 (-0.51, -0.14)*** | 0.09 (0.09) | -0.60 (-0.91, -0.29)*** | 0.12 (0.05) | -0.42 (-0.59, -0.25)*** | 0.16 (0.07) | -0.71 (-0.94, -0.48)*** |
|  | Tofacitinib 10 mg BID | 0.09 (0.06) | -0.25 (-0.44, -0.07)** | 0.09 (0.09) | -0.60 (-0.90, -0.29)*** | -0.03 (0.05) | -0.57 (-0.74, -0.40)*** | -0.11 (0.07) | -0.98 (-1.21, -0.76)*** |
|  | PBO^a^ or MTX^b^ | 0.34 (0.08) | - | 0.68 (0.13) | - | 0.54 (0.07) | - | 0.87 (0.10) | - |
| 7 | Tofacitinib 5 mg BID | 0.00 (0.05) | -0.33 (-0.51, -0.16)*** | 0.08 (0.09) | -0.61 (-0.90, -0.31)*** | 0.09 (0.05) | -0.49 (-0.65, -0.33)*** | 0.16 (0.07) | -0.70 (-0.93, -0.48)*** |
|  | Tofacitinib 10 mg BID | 0.07 (0.05) | -0.27 (-0.44, -0.09)** | 0.07 (0.09) | -0.62 (-0.91, -0.33)*** | -0.03 (0.05) | -0.61 (-0.77, -0.45)*** | -0.12 (0.07) | -0.98 (-1.20, -0.76)*** |
|  | PBO^a^ or MTX^b^ | 0.34 (0.08) | - | 0.69 (0.13) | - | 0.58 (0.07) | - | 0.86 (0.10) | - |
| 8 | Tofacitinib 5 mg BID | 0.00 (0.05) | -0.32 (-0.48, -0.16)*** | 0.08 (0.08) | -0.57 (-0.84, -0.30)*** | 0.08 (0.05) | -0.43 (-0.58, -0.27)*** | 0.14 (0.07) | -0.69 (-0.90, -0.47)*** |
|  | Tofacitinib 10 mg BID | 0.07 (0.05) | -0.25 (-0.41, -0.09)** | 0.07 (0.08) | -0.58 (-0.85, -0.32)*** | -0.03 (0.05) | -0.54 (-0.70, -0.39)*** | -0.10 (0.06) | -0.92 (-1.13, -0.71)*** |
|  | PBO^a^ or MTX^b^ | 0.32 (0.07) | - | 0.65 (0.11) | - | 0.51 (0.07) | - | 0.83 (0.09) | - |
| 9 | Tofacitinib 5 mg BID | 0.01 (0.05) | -0.30 (-0.45, -0.15)*** | 0.08 (0.07) | -0.53 (-0.78, -0.29)*** | 0.08 (0.05) | -0.46 (-0.61, -0.31)*** | 0.14 (0.06) | -0.65 (-0.86, -0.45)*** |
|  | Tofacitinib 10 mg BID | 0.07 (0.04) | -0.24 (-0.39, -0.09)** | 0.05 (0.07) | -0.55 (-0.80, -0.31)*** | -0.05 (0.04) | -0.59 (-0.74, -0.45)*** | -0.08 (0.06) | -0.88 (-1.08, -0.68)*** |
|  | PBO^a^ or MTX^b^ | 0.31 (0.06) | - | 0.61 (0.10) | - | 0.54 (0.06) | - | 0.80 (0.09) | - |
| 10 | Tofacitinib 5 mg BID | 0.01 (0.04) | -0.28 (-0.42, -0.14)*** | 0.07 (0.07) | -0.50 (-0.72, -0.28)*** | 0.11 (0.04) | -0.42 (-0.57, -0.28)*** | 0.10 (0.06) | -0.67 (-0.86, -0.47)*** |
|  | Tofacitinib 10 mg BID | 0.06 (0.04) | -0.22 (-0.36, -0.09)** | 0.05 (0.07) | -0.52 (-0.74, -0.30)*** | -0.04 (0.04) | -0.58 (-0.72, -0.44)*** | -0.11 (0.06) | -0.88 (-1.07, -0.68)*** |
|  | PBO^a^ or MTX^b^ | 0.29 (0.06) | - | 0.57 (0.09) | - | 0.54 (0.06) | - | 0.76 (0.08) | - |

*p<0.05; **p<0.01; ***p<0.001

^a^ORAL Scan; ^b^ORAL Start

BID, twice daily; CI, confidence interval; LS, least squares; mTSS, van der Heijde modified total Sharp score; MTX, methotrexate; PBO, placebo; SE, standard error
